# External Validation of a Risk Stratification Score for B3 Breast Lesions Detected at Ultrasound Core Needle Biopsy

**DOI:** 10.3390/diagnostics10040181

**Published:** 2020-03-26

**Authors:** Cristina Grippo, Pooja Jagmohan, Paola Clauser, Panagiotis Kapetas, Arthur Meier, Annabel M. Stöger, Anna D’Angelo, Pascal A. T. Baltzer

**Affiliations:** 1 Dipartimento di Diagnostica per Immagini, Radioterapia Oncologica ed Ematologia, Istituto di Radiologia, Fondazione Policlinico Universitario A.Gemelli IRCCS, Università Cattolica del Sacro Cuore, 00168 Roma, Italia; cris.grippo@gmail.com (C.G.); anna.dangelo05@gmail.com (A.D.); 2 Department of Diagnostic Imaging, National University Hospital and Yong Loo Lin School of Medicine, National University of Singapore, Singapore 117597, Singapore; poojajagmohan@gmail.com; 3 Department of Biomedical Imaging and Image-Guided Therapy, Medical University and General Hospital of Vienna, Waehringer Guertel 18-20, A-1090 Vienna, Austria; panagiotis.kapetas@meduniwien.ac.at (P.K.); meier_arthur@web.de (A.M.); annistoeger@gmail.com (A.M.S.); pascal.baltzer@meduniwien.ac.at (P.A.T.B.)

**Keywords:** ROC curve, clinical decision-making, breast, sensitivity and specificity, ultrasound-guided core needle biopsy

## Abstract

Objective: The aim of this study was to externally validate the feasibility and robustness of a risk-stratification score for B3 lesions based on clinical, pathological, and radiological data for improved clinical decision making. Methods: 129 consecutive histologically confirmed B3 lesions diagnosed at ultrasound-guided biopsy at our institution were included in this retrospective study. Patient- and lesion-related variables were independently assessed by two blinded breast radiologists (R1, R2), by assigning each feature a score from 0 to 2 (maximum sum-score of 5). Sensitivity, specificity, positive and negative predictive values were calculated at two different thresholds (≥1 and 2). Categorical variables were compared using Chi-squared and Fisher exact tests. The diagnostic accuracy of the score to distinguish benign from malignant B3 lesions was assessed by receiver operating characteristic (ROC) analysis. Results: Surgery was performed on 117/129 (90.6%) lesions and 11 of these 117 (9.4%) lesions were malignant. No cancers were found at follow-up of at least 24 months. Area under the ROC-curve was 0.736 (R1) to 0.747 (R2), with no significant difference between the two readers (*p* = 0.5015). Using a threshold of ≥1, a sensitivity, specificity, PPV, and NPV of 90%/90% (R1/R2), 39%/38% (R1/R2), 11%/12% (R1/R2) and 97%/98% (R1/R2) were identified. Both readers classified 47 lesions with a score ≤1 (low risk of associated malignancy). Of these, only one malignant lesion was underdiagnosed (Ductal carcinoma in situ-G1). Conclusions: In our external validation, the score showed a high negative predictive value and has the potential to reduce unnecessary surgeries or re-biopsies for ultrasound-detected B3-lesions by up to 39%.

## 1. Introduction

Lesions with uncertain malignant potential (B3-lesions) comprise a broad spectrum of histological changes including atypical intraductal epithelial proliferation (AIDEP), consisting of atypical ductal hyperplasia (ADH) and flat epithelial atypia (FEA), lobular neoplasia (LN) including both atypical lobular hyperplasia (ALH) and lobular carcinoma in situ (LCIS), radial scar/complex sclerosing lesions (RS/CSL), papillary lesions (PL), and other lesions such as fibro-epithelial lesions with cellular stroma (FE), and mucocele-like lesions [1]. These histopathological entities represent a relatively small proportion of all image-guided biopsies with prevalence ranging from 5–14%. However, lesions falling into the B3 category constitute a significant challenge for clinical decision making due to the associated risk of malignancy underestimation which varies across different B3 subtypes and can be up to 35% [1,2,3]. While there still is no clear international consensus on how B3 lesions should be managed, the approach to B3 lesions has undergone significant changes in the last decade. Currently, diagnostic surgical excision is no longer the only available management option and percutaneous excision using a vacuum-assisted device, known as a vacuum assisted breast biopsy (VABB), is recommended for many of these pathological entities [4,5]. However, most cases diagnosed at core needle biopsy (CNB) are still referred to surgery to examine the entire lesion and establish a definitive diagnosis [6,7]. While surgical excision for B3 lesions results in an upgrade to malignancy in some patients, it must be considered an overtreatment due to risks and costs in case there is no underlying malignancy [8,9,10]. Given the differing upgrade rates to malignancy, an accurate estimation of the risk to individual patients in case of a newly diagnosed B3 lesion is the key to tailored management that avoids both under and over-treatment. Many studies have investigated imaging and histopathological features that may be useful in predicting the likelihood of malignancy in order to select cases in which surgical excision may not be necessary [11,12]. To date, no clinical or radio-pathological feature alone, or in combination, has been proven to accurately characterize borderline lesions with less than 2% chance of carcinoma at surgical excision, and to be able to be managed with short-term follow-up, according to the American College of Radiology recommendations [13]. Recently, a new risk stratification system for US-detected breast B3 lesions has been published by Giuliani et al. The score, based on clinical, radiologic, and pathologic data, aims to identify low-risk patients for whom a conservative management could be acceptable, and high-risk patients, for whom open surgery is recommended [14]. According to the authors´ results, the score could be a valuable tool in the clinical management of B3 lesions. Nevertheless, external validation is crucial in assessing the utility of a decision algorithm and its applicability in a generalized population [15]. Therefore, the aim of this study was to independently validate the feasibility and robustness of the Giuliani score in an external cohort of patients.

## 2. Methods and Materials

### 2.1. Patient Population

**Figure 1:**
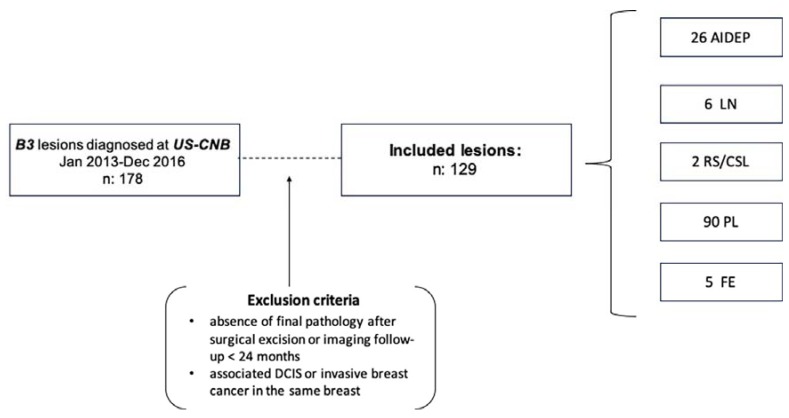
Study outline and final lesions analyzed. US-CNB: ultrasound-guided core needle biopsy. DCIS: ductal carcinomas in situ, AIDEP: atypical intraductal epithelial proliferation, LN: lobular neoplasia, RS/CSL: radial scar/complex sclerosing lesions, PL: papillary lesions, FE: fibro-epithelial lesions with cellular stroma.

The Institutional Review Board granted permission for this retrospective study. Informed consent was obtained from each patient for the biopsy procedure. A systematic review of all the pathologic results of core needle breast biopsies was performed between January 2013 and December 2016 at the Vienna General Hospital, Medical University of Vienna, Austria and 178 consecutive B3 results were retrieved. The inclusion criteria for this retrospective analysis were: (a) histologically confirmed B3 lesions diagnosed on US-guided CNB; (b) diagnostic surgical excision with histopathological examination of the entire lesion; or (c) availability of radiologic follow-up (FU) ≥ 24 months. Exclusion criteria were: (1) synchronous ductal carcinoma in situ (DCIS) or invasive breast cancer in the same breast; (2) absence of final pathology after surgical excision or imaging follow-up <24 months (Figure 1). All histopathology workup was performed by board-certified breast pathologists from the department of pathology at our university hospital in accordance with national S3 guidelines [16]. Forty-nine patients were excluded due to the absence of final pathology or adequate follow-up. A total of 129 B3 lesions in 122 women (115/122 women had one lesion and 7/122 women had two lesions) aged 23–78 years (mean 51.6 years) were included in our study. Of these lesions, 90 (69.8%) were PL, 26 (20.1%) were AIDEP, 6 (4.6%) were LN, 2 (1.6%) were RS/CSL and 5 (3.9%) were FE.

### 2.2. Biopsy Technique

All CNBs were performed using sonographic guidance by one of three experienced breast radiologists with the patient in a supine position, using an Acuson S3000 device (Siemens Medical Solutions, Mountain View, CA, USA) or with an Aplio 500 echograph (Toshiba Medical Systems Corporations, Otawara-shi, Tochigi-ken, Japan), equipped with a 6.2- to 12-MHz and a 5.5- to 18-MHz linear transducer. All lesions were biopsied using a 14G automated biopsy gun (BIP-HistoCore^®^; BIP Medical, Tuerkenfeld, Germany). The exact number of cores obtained varied from patient to patient, with a mean of five specimens per lesion [17].

### 2.3. Data Collection and Analysis

Patient-related and lesion-related variables were collected, following the methodology reported in the original paper [14], including, (a) age (years), (b) lesion type (mass/non-mass), (c) Lesion size (≤10 mm | >10 mm); (d) BI-RADS category, and (f) CNB histological result. For the age of the patients, the cut-off value chosen was 50 years, so the scores were assigned as follows: age <50 years: score 0; age ≥50 years: score 1. Regarding lesion type, even if “non-mass” lesions are not included in the BI-RADS US lexicon, the score considers a US-visible “mass lesion” as a space-occupying lesion, seen on multiple different US images, while a “non-mass lesion” is a hypoechoic area with an indistinct margin [18]. The points assigned were as follows: mass lesion: score 0; non-mass lesion: score 1. Lesion size was routinely assessed according to the maximum lesion diameter, with a threshold of >10 mm for the score 1. Depending on the BI-RADS category assigned to each lesion, the score was assigned as follows: BI-RADS 3: score 0; BI-RADS 4: score 1; BI-RADS 5: score 2. The CNB results were categorized as B3 lesions without atypia (B3a: PL, RS/CSL, FE, and “mucocele-like” lesion) and B3 lesions with atypia (B3b: ADH, FEA, and LIN), as previously published [1,11]. All variables were independently assessed by two off-site fellowship-trained readers experienced in breast imaging (R1, R2), blinded to the final histopathological results. To each variable, a point was assigned according to the score, to a maximum of 5.

### 2.4. Statistical Analysis

Data analysis was performed with commercially available software (IBM SPSS Statistics for Windows version 24.0.2.). The excision histology result or a stable follow-up at imaging >24 months was considered the gold standard for comparison. Statistical evaluation was performed with the Chi-squared and the Fisher exact test for categorical variables. A receiver operating characteristic (ROC) analysis was performed and the area under the ROC curve was measured to determine overall diagnostic performance. Sensitivity, specificity, and likelihood ratios were calculated at different cut-off values with a 95% confidence interval. *p*-values ≤ 0.05 were considered statistically significant.

## 3. Results

### 3.1. Patients and Lesions

**Figure 2:**
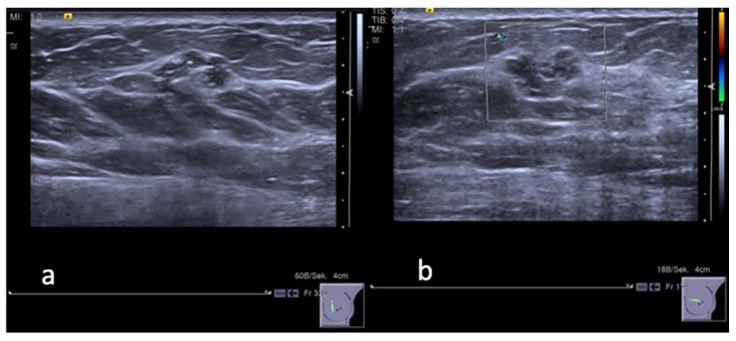
A 48 years old woman. (**a**) Gray-scale ultrasound image shows a hypoechoic «mass» lesion with lobulated and partially non circumscribed margins in the inner para-areolar region of the left breast. The maximum diameter of the lesion was 14 mm. (**b**) Color Doppler image reveals no peripheral vascularity of the lesion. The lesion was categorized by both readers as BI-RADS 3. CNB: B3 lesion without atypia (adenosis, sclerosing adenosis, epithelial proliferation without atypia, small papilloma). Final histology after surgery revealed a highly differentiated, intraductal carcinoma DCIS-G1, in addition to extensive adenosis, small papilloma, and lobular neoplasia.

A total of 129 B3 lesions were identified in US-CNB samples of 122 women (mean age 51.6 years). Core needle biopsy histologic results were the following: 26 (20.1%) atypical intraductal epithelial proliferation (AIDEP), six (4.6%) lobular neoplasia (LN), two (1.6%) radial scar/complex sclerosing lesion (RS/CSL), 90 (69.8%) papillary lesion (PL), and five (3.9%) fibro-epithelial lesions (FE). Surgery was performed on 117/129 (90.6%) B3 lesions, and 11/117 lesions (9.4%) were upgraded to malignant lesions: five ductal carcinomas in situ (DCIS; 4 G1, 1 G2), and six invasive ductal carcinomas not otherwise specified (IDC NOS), with an overall PPV for malignancy of 8% (11/129). Six malignancies (four DCIS, two IDC) were found among 26 lesions diagnosed as AIDEP at CNB (lesion-specific underestimation rate: 23%, 6/26); one (IDC) among six LN (underestimation rate: 16%, 1/6) and one (IDC) among two RS (underestimation rate 50%, 1/2). Among 90 papillary lesions, three malignancies were found, respectively one G1 DCIS (Figure 2) and two IDC, with an underestimation rate of 3% (3/90). None of the FE diagnosed on CNB were associated with malignancy after surgical excision. The remaining 12/129 lesions (9%) were unchanged at imaging FUP after 24 months. Detailed description of histopathological diagnoses is given in Table 1.

### 3.2. ROC Curve Analysis

**Figure 3:**
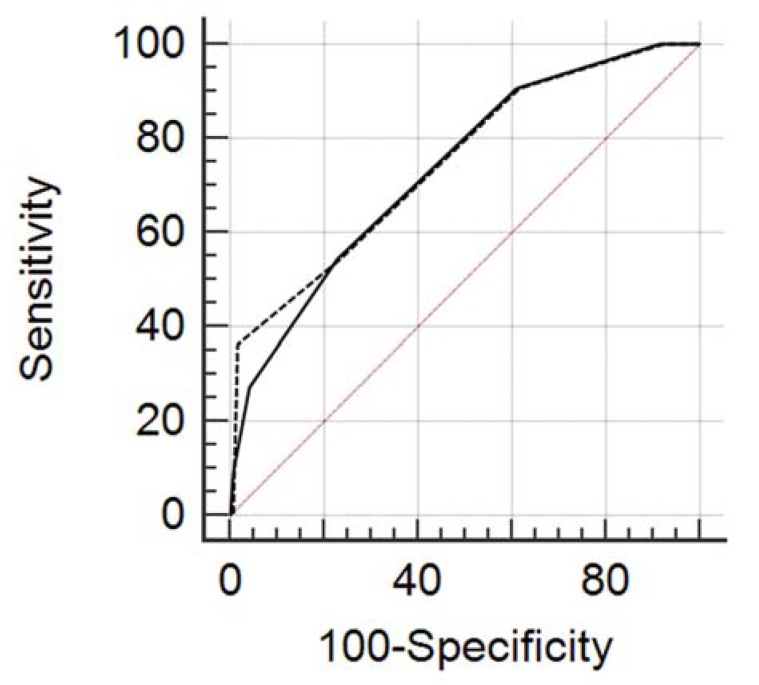
Overall diagnostic accuracy represented by the area under the ROC curve (AUC) ranged from 0.736 (R1—dashed line: 95% CI: 0.662 to 0.819 *p* = 0.0019) to 0.747 (R2—solid line: 95% CI: 0.662 *p* = 0.0018). There was no statistically significant difference between the two readers.

Using the score for our lesions database, the overall diagnostic accuracy represented by the area under the ROC curve (AUC) ranged from 0.736 to 0.747 (R1 95% CI: 0.662 to 0.819, *p* = 0.0019; R2 95% CI: 0.662, *p* = 0.0018) (Figure 3). There was no statistically significant difference between the two readers (AUC R1 = 0.736, standard error = 0.075; AUC R2 = 0.747, standard error = 0.0795; *p* = 0.501). Detailed results of different cut-off levels and their diagnostic parameters in the two readers are shown in Table 2 and Table 3. With a threshold of 2 (low risk: score 0–2; high risk: 3–5), as proposed in the original paper, of 118 benign lesions in our lesions database, 91 (77%) could have been predicted using the score ruling out malignancy, with an NPV of 94.8%. A cut-off of ≤1 to rule out malignancies would have predicted, in both readers, 46/118 true negative with a sensitivity of 90% and an NPV of 98%. Among 47 lesions with a score of ≤1 only one (1/47, 2%) was upgraded at surgical biopsy. This was a case of a PL without atypia characterized by benign features (round shape, microlobulated margins, no intralesional vascularity). In spite of its benign appearance it turned out to be a grade 1 DCIS (Figure 2). Among 82 lesions with a score from 2 to 5, thus considered in the high-risk category, 64 were benign at final histology after surgery, 10 were upgraded to malignancy and 8 were stable at imaging follow-up for longer than 24 months.

## 4. Conclusions

Management of breast B3 lesions has undergone notable change over the last decade. Historically, all B3 lesions were managed with surgical excision, regardless of the underlying lesion type, to exclude any coexisting malignancy. However, for four out of five women with a B3 lesion, this would mean unnecessary surgery [3]. Although recent consensus recommendations suggest a less invasive approach [4,5], management of lesions of uncertain malignant potential still provides a tough challenge to the multidisciplinary team. In this context, the score proposed by Giuliani et al. [14] tried to provide guidance for management recommendations according to the histologic sub-classification in lesion with and without atypia and to other variables that are easily available and commonly used in the clinical practice. The role of these parameters in the prediction of upgrade rate of B3 lesions has already been investigated in other studies [12,19,20,21]. Mean age, among clinical variables, is recognized to be a factor influencing the upgrade rate. A significant difference in the upgrade rate to malignancy has been also described with regard to lesion size (>10 mm) and final BI-RADS 4–5 category assessment [12,21]. In the current study we evaluated the applicability and the robustness of this risk stratification score [14] in an external population. In our series, we found an overall upgraded rate (8%, 11/129) slightly lower than the values in current literature (ranging from 12–35%), and also than the one reported in the paper of Giuliani et al. (21.6%). This could be explained by the high percentage of papillary lesions (90/129, 69%) with a lower lesion-specific PPV (3% vs. 16.2%), probably due to the fact that PL in our series were all lesions without epithelial atypia that usually show lower value of underestimation, between a range of 4 ad 12% [10]. Conversely, the percentage of ADH in our lesions database is very similar to the study population of the original paper, with a slightly lower PPV in our series (23% vs. 33%). It has to be noticed a remarkable difference between the two populations regarding the percentage of RS/CS between two series. Our lesions database consisted of only 2 cases of RS/CSL, thus yielding a lesion-specific upgrade rate of 50%, which is not in agreement with values published in current literature, ranging between 0–16% [22]. The lower percentage of this histopathological entity in our series might be explained by the biopsy method used, in our institution RS/CSL commonly are referred to vacuum-assisted biopsy (VABB) and not to CNB. As in the original paper, in our case series we did not observe any FE upgraded at final histology. In the present study, the score showed a good diagnostic performance (ROC AUC from 0.747 to 0.795) with no significant difference between the two readers (*p* = 0.5015) (Figure 3). Interestingly, using a lower threshold of ≥1 point, the score showed a notably high NPV (98%) thus enabling the detection of low-risk patients that could have avoided surgery. In fact, in at least 47 lesions with a score of 0 or 1, only one was found to be malignant at the final histology and it was a low grade intraductal carcinoma (G1-DCIS). This underlines the potential to use the proposed score for management decisions, specifically to avoid surgery in low risk lesions. Our study has some limitations. First, the retrospective nature of the study, secondly, this study was performed at a single site institution and included a relatively small number of B3 lesions. As a result, some variable subgroups were poorly populated. For 12 lesions of the lesions database, final excision histology was not available. However, volumetric stability at imaging of a lesion for at least 24 months has been previously used as a reliable criterion of benignity [14,23]. In our study, we did not consider the role of contrast-enhanced breast MRI in the evaluation of high-risk lesions. However, even if the negative predictive value of MRI (96–98%) [24,25], might be very useful in identifying patients who could potentially avoid surgery, breast MRI is not easily available in all institutions and its correct interpretation requires skills and expertise that might not be uniformly allocated. In addition, the NPV achieved by using readily available information alone was comparable to the potential NPV provided by MRI. In conclusion, our study validates the applicability and robustness of the proposed risk stratification score. The high NPV might be helpful to avoid unnecessary surgery in B3 lesions in lesions diagnosed at US-CNB that are considered low-risk by the score.

## Figures and Tables

**Table 1:** Descriptive statistics for histopathological diagnoses among all B3 lesions diagnosed ad US-CNB. Final histologic results for each sub-category and associated positive predictive value (PPV) for breast malignancy.

US-CNB	*N*	%	FINAL DIAGNOSIS		PPV
			Non Malignant	Malignant	
**AIDEP**	26	20.1	20	6	23%, 6/26
**LN**	6	4.6	5	1	16%, 1/6
**RS/CSL**	2	1.6	1	1	50%, 1/2
**PL**	90	69.8	87	3	3%, 3/90
**FE**	5	3.9	5	0	0%
**Total**	129	100	118	11	8%, 11/129

*n* number, PPV: positive predictive value, AIDEP: atypical intraductal epithelial proliferation, LN: lobular neoplasia, RS/CSL: radial scar/complex sclerosing lesions, PL: papillary lesions, FE: fibro-epithelial lesions with cellular stroma.

**Table 2:** Diagnostic parameters and cut-off values of the score for reader R1.

All Lesions						
Cut-Off	Sens.	95% CI	Spec.	95% CI	+LR	−LR
**≥0**	100%, 11/11	71.5–100.0	0%, 0/118	0.0–3.1	1.00	
**>0**	100%, 11/11	71.5–100.0	8.47%, 10/118	4.1–15.0	1.09	0.00
**>1**	90.91%, 10/11	58.7–99.8	38.98%, 46/118	30.1–48.4	1.49	0.23
**>2**	54.55%, 6/11	23.4–83.3	77.12%, 91/118	68.5–84.3	2.38	0.59
**>3**	27.27%, 3/11	6.0–61.0	95.76%, 113/118	90.4–98.6	6.44	0.76
**>4**	9.09%, 1/11	0.2–41.3	99.15%, 117/118	95.4–100.0	10.73	0.92
**>5**	0%, 0/11	0.0–28.5	100.00%, 118/118	96.9–100.0		1.00

*n* number, *Sens* sensitivity, *CI* confidence interval, *Spec* specificity, *+LR* positive likelihood ratio, −LR negative likelihood ratio.

**Table 3:** Diagnostic parameters and cut-off values of the score for reader R2.

All Lesions						
Cut-Off	Sens.	95% CI	Spec.	95% CI	+LR	−LR
**≥0**	100%, 11/11	71.5–100.0	0%, 0/118	0.0–3.1	1.00	
**>0**	100%, 11/11	71.5–100.0	7.63%, 9/118	3.5–14.0	1.08	0.00
**>1**	90.91%, 1/11	58.7–99.8	3814%, 45/118	29.4–47.5	1.47	0.24
**>2**	54.55%, 6/11	23.4–83.3	76.27%, 90/118	67.6–83.6	2.30	0.60
**>3**	36.36%, 4/11	10.9–69.2	98.31%, 116/118	94.0–99.8	21.45	0.65
**>4**	0%, 0/11	0.0–28.5	99.15%, 117/118	95.4–100.0	0.00	1.01
**>5**	0%, 0/11	0.0–28.5	100%, 118/118	96.9–100.0		1.00

*n* number, *Sens* sensitivity, *CI* confidence interval, *Spec* specificity, *+LR* positive likelihood ratio, −LR negative likelihood ratio.

## Abbreviations

AIDEP	atypical intraductal epithelial proliferation
ADH	atypical ductal hyperplasia
FEA	flat epithelial atypia
LN	lobular neoplasia
ALH	atypical lobular hyperplasia
LCIS	lobular carcinoma in situ
RS/CSL	radial scar/complex sclerosing lesions
PL	papillary lesions
FE	fibro-epithelial lesions with cellular stroma
VABB	vacuum assisted breast biopsy
US-CNB	ultrasound guided core needle biopsy
ROC	receiver operating characteristic
AUC	area under the curve
NPV	negative predictive value
PPV	positive predictive value
DCIS	ductal carcinoma in situ
